# Contemporary Role of Positron Emission Tomography (PET) in Endocarditis: A Narrative Review

**DOI:** 10.3390/jcm13144124

**Published:** 2024-07-15

**Authors:** Antonio Maria Sammartino, Giovanni Battista Bonfioli, Francesco Dondi, Mauro Riccardi, Francesco Bertagna, Marco Metra, Enrico Vizzardi

**Affiliations:** 1 Institute of Cardiology, ASST Spedali Civili di Brescia, Department of Medical and Surgical Specialties, Radiological Sciences and Public Health, University of Brescia, 25121 Brescia, Italy; g.bonfioli@unis.it (G.B.B.);; 2 Nuclear Medicine, ASST Spedali Civili di Brescia, Department of Medical and Surgical Specialties, Radiological Sciences and Public Health, University of Brescia, 25121 Brescia, Italy

**Keywords:** ^18^F-FDG, PET, endocarditis, nuclear imaging, CIED, valvular cardiac infection

## Abstract

Endocarditis, a serious infectious disease, remains a diagnostic challenge in contemporary clinical practice. The advent of advanced imaging modalities has contributed significantly to the improved understanding and management of this complex disease. 18F-fluorodeoxyglucose (18F-FDG) positron emission tomography (PET) imaging has shown remarkable potential in improving the diagnostic accuracy of endocarditis. In the update of the Modified Duke Criteria, in 2023, The International Society for Cardiovascular Infectious Diseases (ISCVID) Working Group recognized specific 18F-FDG PET/CT findings as a major diagnostic criterion, particularly in patient with prosthetic valve endocarditis. The ability of PET to visualize metabolic activity allows for the identification of infective foci and could differentiate between infective and non-infective processes. This review examines the clinical utility of PET in differentiating infective endocarditis from other cardiovascular pathologies, highlighting its sensitivity and specificity in detecting native and prosthetic valve infections, including patients with transcatheter aortic valve implantation (TAVI), cardiac implantable devices (CIEDs), and left ventricular assistance devices (LVAD). Also, practical aspects and indications are illustrated to optimize the quality of imaging and reduce potential false positive results. In conclusion, the current use of PET in endocarditis has become a valuable diagnostic tool; as technological advances continue, PET will play an increasingly important role in the multidisciplinary approach to the management of endocarditis.

## 1. Introduction

Infective endocarditis (IE) is a life-threatening cardiac infection with an incidence of ~3–10 per 100,000 people and remains a highly lethal disease with an overall mortality of about 25%. Individuals harboring prosthetic heart valves or implanted cardiac devices may exhibit an increased susceptibility to infectious complications [1,2].

Despite the improvements in diagnostic and therapeutic strategies, the incidence and the severity of IE remains unchanged due to numerous reasons, including an increasing proportion of older patients with more advanced disease, shifts in the types of germs causing these infections, and more frequent infections in people with artificial heart valves or implanted devices [2].

IE demonstrates a variable clinical presentation, ranging from acute onset to a subacute or chronic course. The diagnosis is based on the modified Duke criteria: the major criteria include either the microbiological evidence of infection by typical microorganisms and/or the documentation of cardiac lesions by imaging techniques. The minor criteria include predisposing conditions, fever, embolic vascular dissemination, immunological phenomena, and microbiological evidence [3,4,5]. In 2023, The International Society for Cardiovascular Infectious Diseases (ISCVID) proposed a revised set of Duke Criteria for infective endocarditis (IE) diagnosis, retaining the original framework of “definite”, “possible”, and “rejected” classifications. However, several major and minor criteria have been modified or added. Notably, a “surgical criterion” has been introduced among the major criteria, joining the existing “microbiological” and “imaging” criteria.

Within the major imaging criteria, computed tomography (CT) and positron emission tomography/computed tomography (PET/CT) have gained greater prominence, with PET/CT now recognized as a major diagnostic criterion [6].

## 2. Imaging for the Diagnosis in Endocarditis

The role of imaging is fundamental in the diagnostic definition of infective endocarditis.

Driven by accumulating evidence, a growing population with implanted cardiac electronic devices (CIED) (such as pacemakers and defibrillators), and a subsequent rise in device-related infections, the ISCVID has acknowledged the increasing importance of these factors in infective endocarditis (IE) diagnosis. This recognition extends to the rising incidence of endocarditis on transcatheter-implanted valve prostheses (e.g., transcatheter aortic valve implantation [TAVI] and transcatheter edge-to-edge repair), which now demonstrates a comparable prevalence to surgically implanted prosthetic valve infections. To reflect these evolving trends, the new consensus document for updating the Duke criteria incorporates new imaging criteria and expands the minor criteria category. Notably, the “endovascular cardiac implantable electronic devices” criterion has been added to the list of predisposing factors, joining established categories such as prosthetic heart valves and injection drug use, congenital heart disease, hypertrophic cardiomyopathy, previous valve repair, mild or more severe valve regurgitation, and stenosis [6]. 

In the first instance, the echocardiogram (transthoracic and/or transesophageal) is routinely used for an initial clinical suspicion. Following definitive diagnosis, a comprehensive evaluation for extra-valvular infectious sequelae (abscesses and fistulae) using CT imaging is crucial for optimal patient management. This evaluation may also include magnetic resonance imaging (MRI) to assess for encephalic embolization, potentially contraindicating a surgical approach. Additionally, PET/CT or single-photon emission tomography/computed tomography (SPECT/CT) with radionuclide leukocyte can be employed to identify distant infectious foci, further corroborating the diagnostic hypothesis [7].

### 2.1. Echocardiography: Transthoracic and Transesophageal

Transthoracic echocardiography (TTE) is the first-line imaging technique for suspected IE. However, usually, TTE is not sufficient, except for isolated right-sided native valve IE with good quality examination and unequivocal findings. Transesophageal echocardiography (TOE) is the key imaging technique both for the diagnosis and for the identification of valvular and perivalvular complications (abscess, pseudoaneurysm, and new partial dehiscence of prosthetic valve) and for the description of vegetation characteristics and size, crucial parameters that guide surgical indication [3,4,7,8,9].

In cases where vegetations are present, echocardiography (both transthoracic and transesophageal) demonstrates a good weapon for the diagnosis of IE with sensitivities of 75% and 85–90%, respectively. However, the diagnostic accuracy is lower in patients with prosthetic valve endocarditis (PVE). While transesophageal echocardiography (TEE) boasts a near-perfect sensitivity of around 99% for diagnosing native valve endocarditis, its effectiveness dips to roughly 90% for Cardiac Implantable Electronic Device (CIED)-related endocarditis [9,10]. 

This decrease in sensitivity underscores the importance of incorporating additional imaging modalities to enhance diagnostic accuracy in CIED-related cases. Other imaging modalities, such as CT, nuclear imaging, and magnetic resonance imaging (MRI), are needed to confirm or exclude the diagnosis of IE and to diagnose eventual extracardiac complications [10]. 

### 2.2. Computed Tomography (CT)

Cardiac CT offers complementary insights beyond echocardiography, making it a valuable tool for targeted patient evaluation in specific clinical scenarios. It excels in evaluating valvular and myocardial anatomy, offering high-resolution capabilities for characterizing coronary arteries (alternative to an angiography), cardiac walls, and valvular planes.

Cardiac CT is indicated to diagnose local complication, such as abscesses, pseudoaneurysms and fistulae, for which it is more accurate than TOE. Furthermore, CT may detect distant lesions that can be relevant in decision making, particularly in the pre-operative assessment [11,12,13,14,15,16].

However, TEE proved superior to cardiac CT in detecting vegetations (94% vs. 64%), valvular perforations (81% vs. 41%), and paravalvular leakage (69% vs. 44%). Compared to transesophageal echocardiography (TEE), cardiac CT demonstrated a higher success rate (78% vs. 69%) in identifying pseudoaneurysms or abscesses [17].

For these reasons, according to the ISCVID Working Group, these two imaging modalities (echocardiography and cardiac CT) offer a combined approach for the diagnosis of IE.

### 2.3. Magnetic Resonance Imaging (MRI)

Cardiac magnetic resonance imaging (MRI) has a limited role in the diagnostic context of infectious endocarditis due to its lower spatial resolution compared to CT or PET [18,19].

The recently updated Duke-ISCVID criteria incorporate an expanded list of minor criteria encompassing immunological and vascular manifestations, such as Janeway lesions, Osler’s nodes, and Roth spots. In this context, vascular phenomena include embolic infarcts, mycotic aneurysm and intracranial hemorrhage. These findings, which can be detected using brain imaging techniques, can influence decisions regarding medical or surgical treatment options [17].

In this setting, brain MRI could help in the diagnosis and management of the patient with suspected IE; it plays a crucial role in the identification of ischemic brain lesions [20,21,22], with an accuracy of 89–94% in the diagnosis of spondylodiscitis or osteomyelitis, as possible distant foci of infection [23,24,25]. 

Ahn et al. investigated the impact of brain MRI on the diagnosis of infective endocarditis. Specifically, they employed a systematic review and a meta-analysis to evaluate the clinical impact of brain MRI on the diagnosis of infective endocarditis. Their analysis found 2133 patients with infective endocarditis with 1399 lesions: acute ischemic lesions were the most frequent (61.9%), followed by cerebral microbleeds (52.9%), brain abscess or meningitis (9.5%), and intracranial mycotic aneurysm (6.2%) [26].

They also investigated the role of MRI brain findings in clinical decision. Their analysis also included studies that evaluated changes in endocarditis diagnosis from “possible” to “definite”, modifications in therapy, and alterations in surgical planning as outcomes. Two studies reported “diagnosis modification” rates of 5.4% and 32.1%, respectively, with a shift to “definite IE” in 16 patients previously classified as “possible IE”. Therapy modification was documented for a total of 43 patients (12.8%, 95% CI, 6.5–23.7%). This included the initiation of anticoagulant therapy, the commencement of antifungal therapy and, in two patients, the need for neurosurgical intervention in some patients. Finally, surgical planning was reassessed in 14.2% (95% CI, 8.2–23.4%) of cases, with a change in indication for cardiac surgery or postponed surgery.

These results suggest that brain MRI screening could be beneficial, even if patients do not have any neurological symptoms.

### 2.4. Nuclear Medicine: Radiolabeled Leukocyte SPECT/CT

Nuclear medicine procedures leverage radionuclide tracers to directly visualize sites of infection-induced inflammatory processes within the body. Most common modalities employed for infection diagnosis are the use of 18F-fluorodeoxyglucose PET/CT (18F-FDG PET/CT) scans and radiolabeled leukocyte SPECT/CT scans using radioactive 99m-Tc or 111In-oxine.

Single-photon emission computed tomography (SPECT)/CT imaging with radiolabeled leukocytes is a well-established diagnostic modality for visualizing infectious processes within the body. This technique offers the advantage of not requiring any specific patient preparation; however, the overall imaging protocol can take one to two days to complete [27].

This technique is great for finding infections, but the low spatial resolution and low signal intensity limit the detection of small infective foci (smaller than 1 cm) and those caused by non-pyogenic germs. For patients suspected of having a heart valve infection caused by pyogenic bacteria, a positive radiolabeled white blood cell SPECT/CT scan is a very important finding. For other types of heart valve infections or infections in implanted heart devices (CIED), this test is great as an extra tool [28,29,30]. 

Despite its utility in visualizing infectious processes, radiolabeled leukocyte SPECT/CT imaging is not included among the major or minor criteria for the modified Duke criteria for infective endocarditis diagnosis. Therefore, while this imaging technique can sometimes be a helpful adjunct for clinicians to corroborate a diagnosis, a positive scan result alone does not constitute a diagnostic criterion, as is the case for 18F-FDG PET/CT.

## 3. Positron Emission Tomography (PET)/CT in Endocarditis

In the novel consensus document, The International Society for Cardiovascular Infectious Diseases (ISCVID) Working Group recognizes specific 18F-FDG PET/CT findings as a major diagnostic criterion, particularly in patients with prosthetic valve endocarditis (PVE). This imaging technique can shift a significant number of suspected PVE cases from the “possible” category to a definitive diagnosis of infective endocarditis (IE). According to the definition of major criteria of imaging, the findings with PET/CT include intense, localized (focal), or multiple (multifocal) areas of FDG uptake with a non-uniform pattern (heterogeneous), appearing at least 3 months after valve replacement surgery [6]. 

In work-up diagnosis, FDG-PET/CT in suspected infective endocarditis plays a dual role:

- Identifying infections within the heart: this includes detecting infections in native valves, prosthetic valves, and implanted cardiac devices. Performance varies depending on the location: sensitivity is low for native valve infections and for infections involving cardiac device leads. Sensitivity is significantly higher for prosthetic valve infections and infections within cardiac device pockets. Specificity remains high across all these scenarios;
- Uncovering hidden infectious spread: FDG-PET/CT can also detect clinically silent disseminated infectious disease, identifying primary infection source, and/or septic embolism, which can be crucial for diagnosis and guiding patient management.

### 3.1. Practical Aspects and Image Acquisition Protocols

The optimal timing of 18F-FDG-PET/CT and patient preparation is important for successful imaging [31,32]. Dietary carbohydrate intake typically stimulates insulin secretion, activating the predominantly expressed glucose transporter GLUT4 in normal myocardium and facilitating glucose entry into cells. In the absence of carbohydrates and insulin, myocardial energy metabolism shifts to free fatty acids [31]. The recommendation of the Society of Nuclear Medicine and Molecular Imaging (SNMMI)/American Society of Nuclear Cardiology (ASNC)/Society of Cardiovascular CT (SCCT) and European Association of Cardiovascular Imaging (EACVI) and the European Association of Nuclear Medicine (EANM) suggests a high-fat, low-carbohydrate (HFLC) diet for 12–24 h with fasting for 12–18 h prior to the scan to enhance the optimal suppression of physiological myocardial glucose metabolism. Furthermore, it is recommended to inject the tracer when the blood glucose is <11 mmol/L, or <180 mg/dL [12,33,34,35,36].

According to routine protocols, acquisition is usually performed 45–60 min after the intravenous injection of 18F-FDG. It is known that different uptake times can lead to different intensity of tracer uptake, and this fact has been suggested as able to enhance the diagnostic ability of these imaging modalities in different clinical settings. In their study, even if with only 27 patients, Leccisotti et al. suggests that delayed imaging acquired 3 h after injection is associated with greater accuracy for diagnosing pacing lead infections (70% vs. 51%, *p* = 0.024) in comparison with the standard protocol (1 h after) [37]. 

Moreover, in addition to diet, some studies aimed at optimizing preparation have suggested intravenous heparin administration (to promote lipolysis and the availability of free fatty acids) [38]. The intravenous injection of these molecules before the PET/CT scan has been used alone or in combination with high-fat/low-carbohydrate diet preparation to suppress myocardial uptake; however, the effect of heparin on myocardial glucose uptake has been unclear and inconsistent [39].

As mentioned, different conditions can influence the ratio of tracer uptake when performing a PET/CT study. Antimicrobial treatment is considered to decrease the intensity of 18F-FDG accumulation; however, currently, the discontinuation of the treatment is not recommended before performing PET/CT [40]. Regarding this issue, Swart et al. found that FDG PET/CT was positive in 13 of 28 (46%) patients despite a low level of inflammation (C-reactive protein < 40 mg/L) during the empirical phase of antibiotic therapy [41].

Steroid treatment should be discontinued or reduced to the lowest possible dose in the 24 h preceding the examination [42]. The processing and the creation of PET images is based on different reconstruction protocols and also on the correction of the attenuation of the photons emitted by the tracer by using a CT. Due to the density of implantable cardiac devices and prosthetic valves, artifacts such as beam hardening and scatter may occur and affect this correction, thus making the interpretation of these studies more difficult. In this setting, when a positive and suspected focus of uptake is demonstrated on the aforementioned devices, the use of uncorrected images is mandatory to confirm the diagnosis [43].

One of the added values of PET/CT imaging is its ability to quantify the amount of tracer uptake of different tissues and organs. Semiquantitative analysis employing the standardized uptake value (SUV) is a feasible technique that is widely used in a high number of pathologies to help in the diagnosis, the staging, and the assessment of response to therapy. 

In this setting, the validation of SUV and other semiquantitative PET/CT parameters in inflammation and infection remains, however, unattested. 

Once again, in their large multicenter study (289 patients with prosthetic valve), Swart et al. investigate the capability to identify and exclude potential confounders using EARL-standardized quantitative assessments. They found impressive cutoffs for diagnostic performance in patients with suspected PVE: SUV_max_ ≥ 3.3 sensitivity/specificity of 97%/79%; SUV_ratio_ ≥ 2.0. With a sensitivity/specificity of 100%/91% [41], these results were not influenced by the recent implantation of the prosthetic valves. In clinical practice, the efficacy of quantitative parameters (such as SUVmax normalized to the blood pool activity) between infected and non-infected foci remains controversial.

Finally, an additional promising role of 18F-FDG PET/CT is in patients with established IE, in whom it can be used to monitor response to antimicrobial treatment, transitioning to an alternative therapeutic approach, or determining when treatment can be safely discontinued. This modality is already established for similar purposes in various oncological and inflammatory diseases, and emerging evidence suggests its potential applicability in monitoring the treatment of invasive infections. Despite this, there is currently a lack of data on the use of 18F-FDG PET/CT for treatment monitoring in IE. Given the significant challenges in IE treatment, more research is needed to investigate the value of 18F-FDG PET/CT in this context.

### 3.2. Diagnostic Accuracy and Limitations

Whole-body 18F-FDG PET/CT imaging may be useful to identify infective foci that may be secondary to IE embolization (typically located in the spleen, liver, kidney, muscle and/or vertebral bone in left-side IE or lung in right-side IE) or, on the other hand, the portal of entry of the infection. Due to high physiological uptake of FDG in the brain, PET is less suited to detect cerebral septic embolism [44,45,46,47,48]. In the context of infective endocarditis and cardiac implantable electronic device (CIED) infection, the identification of septic emboli influences the Duke score, thereby impacting diagnostic certainty. 

18F-FDG-PET/CT may also be used to monitor the response to antimicrobial treatment in patients with established IE [49], without indication or not suitable for surgery, that need long-term suppressive antibiotic treatment; however, more prospective studies are needed to evaluate this aspect, as this method can often lead to many false negatives during antibiotic therapy [41,50]. 

The utilization of nuclear medicine imaging shows varying sensitivity, specificity, and accuracy in making a diagnosis, depending on the substrate of infection, whether it is a native valve, prosthetic valve, or implantable device (PM, ICD).

### 3.3. 18F-FDG-PET/CT in Native Valve Endocarditis (NVE)

**Figure 1:**
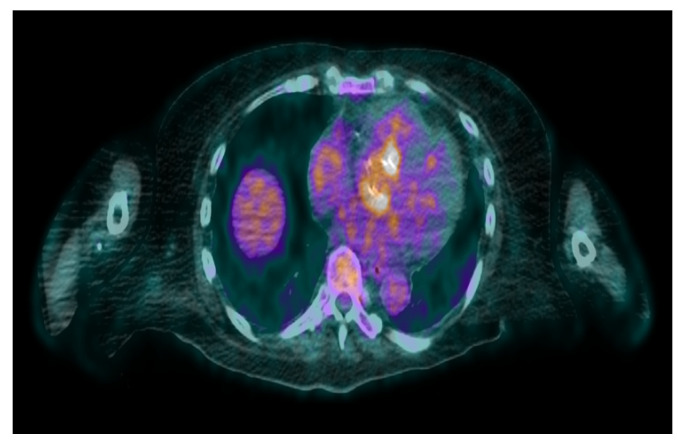
Native aortic valve infection. 18F-FDG PET/CT fused image shows intense uptake of the radiotracer (18F-FDG) localized to the aortic valve of the patient. Diagnosis via transesophageal echocardiography was challenging due to the extensive fibrocalcifications of the aortic valve apparatus in an elderly patient (male, 82 years old).

In patients with native valve, any focal radionuclide uptake should be considered abnormal. However, the presence of valve vegetations in NVE may reduce inflammatory response in favor of a fibrotic reaction, thus lowering the 18F-FDG uptake; therefore, the absence of uptake in this setting cannot exclude the diagnosis of IE [51,52]. Furthermore, NVEs are typically smaller in size compared with prosthetic valve endocarditis (PVE) and consequently may not even be detected by 18F-FDG-PET [51,53]. In fact, in this setting, PET/CT has a low sensitivity (about 31%) but a higher specificity (around 98%) [Figure 1].

The lower sensitivity of 18F-FDG-PET/CT is offset by other strengths of the technique, such as the capability to identify septic emboli and extracardiac manifestations of the infection [46,47].

### 3.4. 18F-FDG-PET/CT in Prosthetic Valve Endocarditis

**Figure 2:**
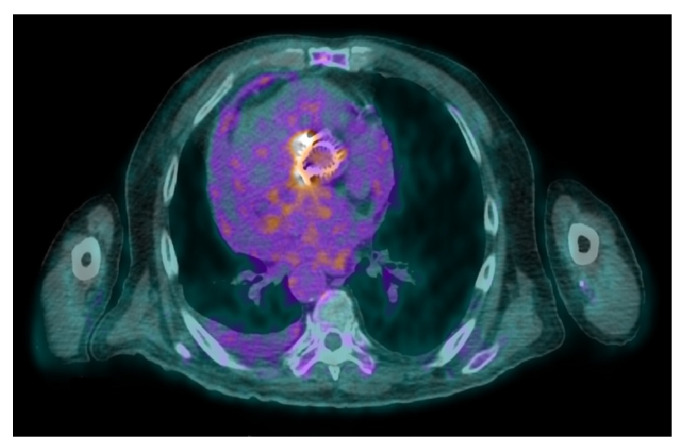
Aortic prosthetic valve infection. 18F-FDG PET/CT fused image demonstrates intense radiotracer uptake at the level of the patient’s aortic valve prosthesis (male, 73 years old).

In prosthetic valve endocarditis (PVE), meta-analysis showed 86% sensitivity and 84% specificity for 18F-FDG-PET/CT [54]. Moreover, 18F-FDG PET/CT improved the sensitivity of the modified Duke criteria from 52–70% to 91–97% [55] by reducing the number of possible prosthetic valve endocarditis (PVE) cases [56,57,58,59,60] [example in Figure 2—prosthetic aortic valve infection].

The studies of Roque [61] and Wahadat [62] have demonstrated that 18F-FDG-PET can also be used in suspected infections of recently implanted prosthetic valves (<1 year); in their studies, the accumulation of tracer at the prosthetic level shows a characteristic pattern of post-operative inflammation, which could be considered a normal finding, if no suspected lesions were described.

Furthermore, Roque et al. suggested Normality Criteria according to the following criteria: qualitative (distribution of 18F-FDG), quantitative (intensity of accumulation), and anatomical (suggestive lesions of IE).

The usefulness of PET/CT has also been studied in the context of patients undergoing transvalvular aortic bioprosthesis implantation (TAVI). A multi-center retrospective study assessed how the addition of FDG-PET/CT, combined with cardiac CT, impacted diagnosis in 30 patients with rejected and possible IE (according to Duke’s criteria) with transcatheter aortic valve implantation (TAVI). Notably, the diagnosis changed (either confirmed or ruled out) for one-third (33%) of the patients thanks to the inclusion of PET scans in their evaluation [63]. Moreover, San et al. studied the 18F-FDG uptake with PET scan after TAVI in non-infected patients. 18F-FDG accumulation was comparable between healthy individuals (controls) and patients with confirmed infective endocarditis (definite IE-TAVI) after transcatheter aortic valve implantation (TAVI). However, the distribution pattern of FDG uptake proved to be a key differentiator [64]. In healthy controls, the pattern was typically uniform (circumferential or hemi-circumferential), while in confirmed IE-TAVI patients, it was often irregular and appeared in isolated spots (focal) or in multiple locations (multifocal). Their study suggests also that, analyzing the pattern of FDG accumulation, FDG-PET/CT can be a reliable tool for diagnosing infective endocarditis (IE-TAVI) at least one month after TAVI procedures.

A distinct subgroup of this setting (cardiac devices) is represented by patients undergoing combined prosthetic aortic valve implantation and ascending aorta replacement, commonly referred to as “Bentall procedure”. Limited information is available regarding 18F-FDG-PET/CT findings for these indications. A focal pattern of the uptake of 18F-FDG or tracer uptake with soft tissue extension may identify suspected Bentall infection with a sensitivity of 86% and a specificity of 80%, as reported in a study enrolling 39 patients [65]. However, more data are needed in this specific setting of patients.

### 3.5. 18F-FDG-PET/CT in Cardiac Implantable Devices and Leads Infections

Inaccurate diagnoses of cardiac device infections pose a double-edged sword. Missed infections (underdiagnosis) are linked to higher mortality due to the risk of sepsis and septic shock, if the pacing system is left in place. Conversely, unnecessary diagnoses (overdiagnosis) can lead to excessive device extraction, resulting in longer hospitalization and potentially increased mortality due to the high-risk procedure. The main manifestations are erythema and abscess formation over pacemakers (PM) or cardioverter defibrillators (ICD) pocket; the infection can extend to the PM/ICD leads or to cardiac valve leaflets. When vegetations on the leads are evident, the pacing system may be removed; without definitive evidence, the management is very challenging.

**Figure 3:**
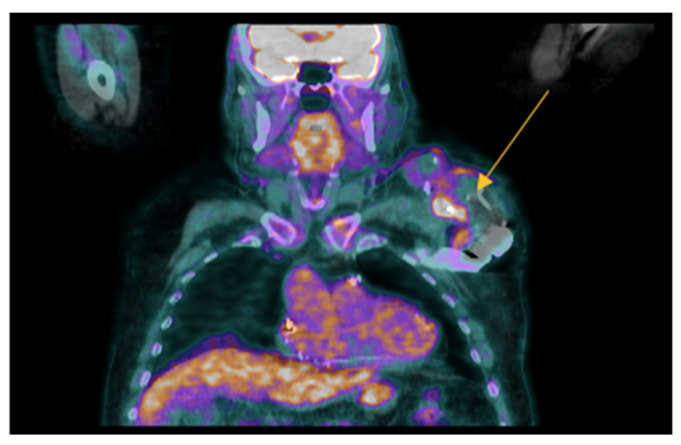
Pacemaker pocket infection. In this case, we reported an image of a 18F-FDG PET/CT scan showing intense uptake at the patient’s pacemaker pocket (yellow arrow) (male, 76 years old).

The incidence of infections involving electronic devices is arising and varies from 0.13% to 19.9% [66,67]. 18F-FDG PET/CT may be useful for the identification of infection of generator/pocket infection (sensitivity 93–96% and specificity 97–98%) more than for the infection of the electro-catheters (sensitivity 65–76% and specificity 83–88%) because of the smaller size of the vegetations and their lower metabolic activity [68,69,70] [Figure 3].

Left ventricular assist devices’ (LVADs’) infections are difficult to diagnose with conventional imaging; in a recent meta-analysis involving patients with suspected left ventricular assist device (LVAD) infection, a total of 256 18F-FDG-PET/CT scans were acquired, and the findings indicated a pooled sensitivity of 97% and 97% and specificity of 99% and 93% for infections of the driveline and of the central device components, respectively [71]. Beyond its ability to diagnose infections, FDG-PET/CT scans were also linked to patient outcomes in a small study of 35 LVAD patients [72]. In most of these patients (28), the FDG-PET/CT was carried out because a device infection was suspected, and the severity of infection detected by the PET/CT scan was connected to the prognosis of patients. This link was further supported by another study involving a larger group of 57 patients who underwent a total of 85 FDG-PET/CT scans [73]. This study showed a tendency for lower survival rates when the scan revealed involvement of all the LVAD components and the lymph nodes in the chest.

These results underline the potential utility of 18F-FDG-PET/CT also in this context; however, larger prospective studies are warranted to further confirm these findings.

## 4. Future Directions

Artificial intelligence (AI) and machine learning (ML) methodologies are being progressively integrated into the realm of nuclear medicine. While significant advancements have primarily been documented in oncology, there is burgeoning interest in exploring their potential applicability in other domains. An intriguing avenue for future investigations would involve assessing whether AI approaches can discern between physiological uptake, reactive inflammation, postsurgical changes, and infection in suspected cases of infective endocarditis (IE). 

In this setting, a recent paper by Godefroy et al. investigated the value of 18F-FDG-based radiomics and ML in the aortic prosthetic valve infective endocarditis diagnosis, revealing that the ML algorithm achieved high diagnostic performance results, particularly when included in the ESC criteria [74].

Currently, 18F-FDG stands as the only PET radiotracer utilized in clinical practice for assessing IE. However, the ongoing evaluation of new radiotracers with bacteria-specific uptake holds promise for substantially enhancing PET/CT diagnostic accuracy in this regard. A systematic review conducted by Auletta et al. explored several potential bacteria-specific candidates, including 18F-Fluorosorbitol, 18F-Fluoromaltohexoase, and 11C-Labeled para-aminobenzoic acid (PABA); it is important to note that they have solely undergone validation in animal models thus far [75,76,77,78,79,80].

## 5. Conclusions

The utility of 18F-FDG PET/CT imaging in the diagnosis and management of patients with confirmed or suspected endocarditis is well-established; an additional diagnostic modality has been demonstrated in assessing complex cases of IE, exhibiting high overall specificity, and in complementary echocardiography and cardiac CT scans.

18F-FDG PET/CT imaging requires specific dietary adjustments beforehand to ensure the most accurate results to diagnosing.

This imaging technique offers greater diagnostic accuracy in suspected prosthetic valve endocarditis (PVE) and infections of implanted cardiac electronic devices (CIED); in PVE and CIED, the sensitivity of echocardiography may be suboptimal with equivocal findings. In contrast, in NVE, 18F-FDG PET/CT had low sensitivity and, therefore, may be of limited diagnostic value.

Moreover, for potential endocarditis diagnoses that were initially suspected based on the Duke criteria, as indicated in the Modified Duke Criteria update of 2023, CT/PET imaging can help to definitively confirm or refute the suspicious of IE. This is important because it can help clinicians make more informed decisions about the treatment and management of patients, especially in complex scenarios with suspected septic emboli or equivocal surgical indications. Looking towards the future, we anticipate advancements in image optimization techniques, potentially leveraging machine learning and artificial intelligence, to further enhance diagnostic accuracy. Additionally, there is hope for harnessing imaging modalities (PET/CT or even SPECT/CT with radiolabeled leukocytes) to gain insights into antibiotic therapy response in endocarditis. Moreover, the development of novel radiotracers with simplified preparation procedures and high diagnostic accuracy holds promise. Further prospective studies are warranted to validate and refine 18F-FDG PET/CT diagnostics, particularly in patient settings where diagnostic accuracy remains suboptimal. These efforts should focus on enhancing sensitivity and specificity, addressing the limitations that currently hinder broader clinical adoption.

While imaging modalities play a valuable and indispensable role in the diagnostic process, clinical suspicion derived from patient history and clinical presentation remains paramount. This underscores the importance of a comprehensive diagnostic approach that integrates both imaging and clinical evaluation, thereby optimizing resource allocation and minimizing unnecessary testing.

## References

[1] Chen H., Zhan Y., Zhang K., Gao Y., Chen L., Zhan J., Chen Z., Zeng Z. (2022). The Global, Regional, and National Burden and Trends of Infective Endocarditis From 1990 to 2019: Results From the Global Burden of Disease Study 2019. Front. Med..

[2] Habib G., Erba P.A., Iung B., Donal E., Cosyns B., Laroche C., Popescu B.A., Prendergast B., Tornos P., Sadeghpour A. (2019). Clinical presentation, aetiology and outcome of infective endocarditis. Results of the ESC-EORP EURO-ENDO (European infective endocarditis) registry: A prospective cohort study. Eur. Heart J..

[3] Baddour L.M., Wilson W.R., Bayer A.S., Fowler V.G., Tleyjeh I.M., Rybak M.J., Barsic B., Lockhart P.B., Gewitz M.H., Levison M.E. (2015). Infective Endocarditis in Adults: Diagnosis, Antimicrobial Therapy, and Management of Complications. Circulation.

[4] Delgado V., Delgado V., Marsan N.A., Marsan N.A., de Waha S., de Waha S., Bonaros N., Bonaros N., Brida M., Brida M. (2023). 2023 ESC Guidelines for the management of endocarditis. Eur. Heart J..

[5] Li J.S., Sexton D.J., Mick N., Nettles R., Fowler Jr V.G., Ryan T., Bashore T., Corey G.R. (2000). Proposed modifications to the Duke criteria for the diagnosis of infective endocarditis. Clin. Infect. Dis..

[6] Fowler V.G., Durack D.T., Selton-Suty C., Athan E., Bayer A.S., Chamis A.L., Dahl A., DiBernardo L., Durante-Mangoni E., Duval X. (2023). The 2023 Duke-International Society for Cardiovascular Infectious Diseases Criteria for Infective Endocarditis: Updating the Modified Duke Criteria. Clin. Infect. Dis..

[7] Habib G., Badano L., Tribouilloy C., Vilacosta I., Zamorano J.L., Galderisi M., Voigt J.-U., Sicari R., Cosyns B., Fox K. (2010). Recommendations for the practice of echocardiography in infective endocarditis. Eur. J. Echocardiogr..

[8] Bai A.D., Steinberg M., Showler A., Burry L., Bhatia R.S., Tomlinson G.A., Bell C.M., Morris A.M. (2017). Diagnostic Accuracy of Transthoracic Echocardiography for Infective Endocarditis Findings Using Transesophageal Echocardiography as the Reference Standard: A Meta-Analysis. J. Am. Soc. Echocardiogr..

[9] Erba P.A., Pizzi M.N., Roque A., Salaun E., Lancellotti P., Tornos P., Habib G. (2019). Multimodality Imaging in Infective Endocarditis an Imaging Team Within the Endocarditis Team. Circulation.

[10] Stainback R.F., Estep J.D., Agler D.A., Birks E.J., Bremer M., Hung J., Kirkpatrick J.N., Rogers J.G., Shah N.R., American Society of Echocardiography (2015). Echocardiography in the management of patients with left ventricular assist devices: Recommendations from the American Society of Echocardiography. J. Am. Soc. Echocardiogr..

[11] Chaosuwannakit N., Makarawate P. (2019). Value of cardiac computed tomography angiography in pre-operative assessment of infective endocarditis. J. Cardiothorac. Surg..

[12] Erba P.A., Lancellotti P., Vilacosta I., Gaemperli O., Rouzet F., Hacker M., Signore A., Slart R.H.J.A., Habib G. (2018). Recommendations on nuclear and multimodality imaging in IE and CIED infections. Eur. J. Nucl. Med. Mol. Imaging.

[13] Kim I.-C., Chang S., Hong G.-R., Lee S.H., Lee S., Ha J.-W., Chang B.-C., Kim Y.J., Shim C.Y. (2018). Comparison of Cardiac Computed Tomography with Transesophageal Echocardiography for Identifying Vegetation and Intracardiac Complications in Patients With Infective Endocarditis in the Era of 3-Dimensional Images. Circ. Cardiovasc. Imaging.

[14] Fagman E., Flinck A., Snygg-Martin U., Olaison L., Bech-Hanssen O., Svensson G. (2016). Surgical decision-making in aortic prosthetic valve endocarditis: The influence of electrocardiogram-gated computed tomography. Eur. J. Cardiothorac. Surg..

[15] González I., Sarriá C., López J., Vilacosta I., San Román A., Olmos C., Sáez C., Revilla A., Hernández M., Caniego J.L. (2014). Symptomatic peripheral mycotic aneurysms due to infective endocarditis: A contemporary profile. Medicine.

[16] Parra J.A., Hernández L., Muñoz P., Blanco G., Rodríguez-Álvarez R., Vilar D.R., de Alarcón A., Goenaga M.A., Moreno M., Fariñas M.C. (2018). Detection of spleen, kidney and liver infarcts by abdominal computed tomography does not affect the outcome in patients with left-side infective endocarditis. Medicine.

[17] Oliveira M., Guittet L., Hamon M., Hamon M. (2020). Comparative value of cardiac CT and transesophageal echocardiography in infective endocarditis: A systematic review and meta-analysis. Radiol. Cardiothorac. Imaging..

[18] El Ouazzani J., Jandou I., Thuaire C. (2020). Thrombus or vegetation?Importance of cardiac MRI as a diagnostic tool based on case report and literature review. Ann. Med. Surg..

[19] Dursun M., Yilmaz S., Yilmaz E., Yilmaz R., Onur I., Oflaz H., Dindar A. (2015). The utility of cardiac MRI in diagnosis of infective endocarditis: Preliminary results. Diagn. Interv. Radiol..

[20] Snygg-Martin U., Gustafsson L., Rosengren L., Alsiö A., Ackerholm P., Andersson R., Olaison L. (2008). Cerebrovascular complications in patients with left-sided infective endocarditis are common: A prospective study using magnetic resonance imaging and neurochemical brain damage markers. Clin. Infect. Dis..

[21] Sotero F.D., Rosário M., Fonseca A.C., Ferro J.M. (2019). Neurological Complications of Infective Endocarditis. Curr. Neurol. Neurosci. Rep..

[22] Cooper H.A., Thompson E.C., Laureno R., Fuisz A., Mark A.S., Lin M., Goldstein S.A. (2009). Subclinical brain embolization in left-sided infective endocarditis: Results from the evaluation by MRI of the brains of patients with left-sided intracardiac solid masses (EMBOLISM) pilot study. Circulation.

[23] Kim S.-J., Pak K., Kim K., Lee J.S. (2019). Comparing the Diagnostic Accuracies of F-18 Fluorodeoxyglucose Positron Emission Tomography and Magnetic Resonance Imaging for the Detection of Spondylodiscitis: A Meta-analysis. Spine Phila Pa 1976.

[24] Foreman S.C., Schwaiger B.J., Gempt J., Jungmann P.M., Kehl V., Delbridge C., Wantia N., Zimmer C., Kirschke J.S. (2017). MR and CT Imaging to Optimize CT-Guided Biopsies in Suspected Spondylodiscitis. World Neurosurg..

[25] Ahn Y., Joo L., Suh C.H., Kim S., Shim W.H., Kim S.J., Lee S.-A. (2022). Impact of Brain MRI on the Diagnosis of Infective Endocarditis and Treatment Decisions: Systematic Review and Meta-Analysis. Am. J. Roentgenol..

[26] Ten Hove D., Slart R.H.J.A., Sinha B., Glaudemans A.W.J.M., Budde R.P.J. (2021). 18F-FDG PET/CT in Infective Endocarditis: Indications and Approaches for Standardization. Curr. Cardiol. Rep..

[27] Erba P.A., Glaudemans A.W., Veltman N.C., Sollini M., Pacilio M., Galli F., Dierckx R.A., Signore A. (2014). Image acquisition and interpretation criteria for 99mTc-HMPAO-labelled white blood cell scintigraphy: Results of a multicentre study. Eur. J. Nucl. Med. Mol. Imaging..

[28] de Vries E.F., Roca M., Jamar F., Israel O., Signore A. (2010). Guidelines for the labelling of leucocytes with (99m)Tc-HMPAO. Inflammation/Infection Taskgroup of the European Association of Nuclear Medicine. Eur. J. Nucl. Med. Mol. Imaging.

[29] Roca M., de Vries E.F., Jamar F., Israel O., Signore A. (2010). Guidelines for the labelling of leucocytes with (111)In-oxine. Inflammation/Infection Taskgroup of the European Association of Nuclear Medicine. Eur. J. Nucl. Med. Mol. Imaging.

[30] Bourque J.M., Birgersdotter-Green U., Bravo P.E., Budde R.P., Chen W., Chu V.H., Dilsizian V., Erba P.A., Gallegos Kattan C., Habib G. (2024). 18F-FDG PET/CT and radiolabeled leukocyte SPECT/CT imaging for the evaluation of cardiovascular infection in the multimodality context. J. Nucl. Cardiol..

[31] Sammartino A.M., Falco R., Drera A., Dondi F., Bellini P., Bertagna F., Vizzardi E. (2023). Vascular inflammation and cardiovascular disease: Review about the role of PET imaging. Int. J. Cardiovasc. Imaging.

[32] Depre C., Vanoverschelde J.L.J., Taegtmeyer H. (1999). Glucose for the Heart. Circulation.

[33] Osborne M.T., Hulten E.A., Murthy V.L., Skali H., Taqueti V.R., Dorbala S., DiCarli M.F., Blankstein R. (2017). Patient preparation for cardiac fluorine-18 fluorodeoxyglucose positron emission tomography imaging of inflammation. J. Nucl. Cardiol..

[34] Lee H.Y., Nam H.-Y., Shin S.K. (2015). Comparison of myocardial F-18 FDG uptake between overnight and non-overnight fasting in non-diabetic healthy subjects. Jpn. J. Radiol..

[35] Dorbala S., Di Carli M.F., Delbeke D., Abbara S., DePuey E.G., Dilsizian V., Forrester J., Janowitz W., Kaufmann P.A., Mahmarian J. (2013). SNMMI/ASNC/SCCT guideline for cardiac SPECT/CT and PET/CT 1.0. J. Nucl. Med..

[36] Slart R.H., Glaudemans A.W., Gheysens O., Lubberink M., Kero T., Dweck M.R., Habib G., Gaemperli O., Saraste A., Gimelli A. (2020). Procedural recommendations of cardiac PET/CT imaging: Standardization in inflammatory-, infective-, infiltrative-, and innervation- (4Is) related cardiovascular diseases: A joint collaboration of the EACVI and the EANM: Summary. Eur. Heart J. Cardiovasc. Imaging.

[37] Leccisotti L., Perna F., Lago M., Leo M., Stefanelli A., Calcagni M.L., Pelargonio G., Narducci M.L., Bencardino G., Bellocci F. (2014). Cardiovascular implantable electronic device infection: Delayed vs standard FDG PET-CT imaging. J. Nucl. Cardiol..

[38] Scholtens A.M., Verberne H.J., Budde R.P., Lam M.G. (2016). Additional Heparin Preadministration Improves Cardiac Glucose Metabolism Suppression over Low-Carbohydrate Diet Alone in 18F-FDG PET Imaging. J. Nucl. Med..

[39] Dilsizian V., Budde R.P., Chen W., Mankad S.V., Lindner J.R., Nieman K. (2022). Best practice for imaging cardiac device-related infections and endocarditis: A JACC Cardiovascular Expert Panel Statement. JACC Cardiovasc. Imaging..

[40] Scholtens A.M., Van Aarnhem E.E.H.L., Budde R.P. (2015). Effect of antibiotics on FDG-PET/CT imaging of prosthetic heart valve endocarditis. Eur. Heart J. Cardiovasc. Imaging.

[41] Swart L.E., Gomes A., Scholtens A.M., Sinha B., Tanis W., Lam M.G., van der Vlugt M.J., Streukens S.A.F., Aarntzen E.H., Bucerius J. (2018). Improving the Diagnostic Performance of 18F-Fluorodeoxyglucose Positron-Emission Tomography/Computed Tomography in Prosthetic Heart Valve Endocarditis. Circulation.

[42] Vera P., Ouvrier M.J., Hapdey S., Thillays M., Pesquet A.S., Diologent B., Callonec F., Hitzel A., Edet-Sanson A., Ménard J.F. (2007). Does chemotherapy influence the quantification of SUV when contrast-enhanced CT is used in PET/CT in lymphoma?. Eur. J. Nucl. Med. Mol. Imaging.

[43] Scholtens A.M., Verberne H.J. (2018). Attenuation correction and metal artifact reduction in FDG PET/CT for prosthetic heart valve and cardiac implantable device endocarditis. J. Nucl. Cardiol..

[44] Mikail N., Benali K., Mahida B., Vigne J., Hyafil F., Rouzet F., Le Guludec D. (2018). 18F-FDG-PET/CT Imaging to Diagnose Septic Emboli and Mycotic Aneurysms in Patients with Endocarditis and Cardiac Device Infections. Curr. Cardiol. Rep..

[45] San S., Ravis E., Tessonier L., Philip M., Cammilleri S., Lavagna F., Norscini G., Arregle F., Martel H., Oliver L. (2019). Prognostic Value of 18F-Fluorodeoxyglucose Positron Emission Tomography/Computed Tomography in Infective Endocarditis. J. Am. Coll. Cardiol..

[46] Boursier C., Duval X., Bourdon A., Imbert L., Mahida B., Chevalier E., Claudin M., Hoen B., Goehringer F., Selton-Suty C. (2020). ECG-Gated Cardiac FDG PET Acquisitions Significantly Improve Detectability of Infective Endocarditis. JACC Cardiovasc. Imaging.

[47] Kouijzer I.J., Bleeker-Rovers C.P., Oyen W.J. (2014). 18F-FDG PET/CT for the Detection of Septic Embolisms in Patients with Infectious Endocarditis. J. Nucl. Med..

[48] Orvin K., Goldberg E., Bernstine H., Groshar D., Sagie A., Kornowski R., Bishara J. (2015). The role of FDG-PET/CT imaging in early detection of extra-cardiac complications of infective endocarditis. Clin. Microbiol. Infect..

[49] Bucy L., Erpelding M.L., Boursier C., Lefevre B., Alauzet C., Liu Y., Chevalier E., Huttin O., Agrinier N., Selton-Suty C. (2023). Real world experience of therapeutic monitoring of medically treated prosthetic valve infective endocarditis by ^18^F-FDG-PET/CT. J. Nucl. Cardiol..

[50] Camazon N.V., Mateu L., Cediel G., Escolà-Vergé L., Fernández-Hidalgo N., Ferrer M.G., Rodriguez M.T.P., Cuervo G., Aragón R.N., Llibre C. (2021). Long-term antibiotic therapy in patients with surgery-indicated not undergoing surgery infective endocarditis. Cardiol. J..

[51] Albano D., Dondi F., Gazzilli M., Giubbini R., Bertagna F. (2021). Meta-Analysis of the Diagnostic Performance of 18F-FDG-PET/CT Imaging in Native Valve Endocarditis. JACC Cardiovasc. Imaging.

[52] de Camargo R.A., Bitencourt M.S., Meneghetti J.C., Soares J., Gonçalves L.F.T., Buchpiguel C.A., Paixão M.R., Felicio M.F., Soeiro A.d.M., Strabelli T.M.V. (2020). The Role of 18F-Fluorodeoxyglucose Positron Emission Tomography/Computed Tomography in the Diagnosis of Left-sided Endocarditis: Native vs Prosthetic Valves Endocarditis. Clin. Infect. Dis..

[53] Pelletier-Galarneau M., Abikhzer G., Harel F., Dilsizian V. (2020). Detection of Native and Prosthetic Valve Endocarditis: Incremental Attributes of Functional FDG PET/CT over Morphologic Imaging. Curr. Cardiol. Rep..

[54] Wang T.K.M., Sanchez-Nadales A., Igbinomwanhia E., Cremer P., Griffin B., Xu B. (2020). Diagnosis of Infective Endocarditis by Subtype Using 18F-Fluorodeoxyglucose Positron Emission Tomography/Computed Tomography: A Contemporary Meta-Analysis. Circ. Cardiovasc. Imaging.

[55] Gomes A., Glaudemans A.W.J.M., Touw D.J., van Melle J.P., Willems T.P., Maass A.H., Natour E., Prakken N.H.J., Borra R.J.H., van Geel P.P. (2017). Diagnostic value of imaging in infective endocarditis: A systematic review. Lancet Infect. Dis..

[56] Pizzi M.N., Dos-Subirà L., Roque A., Fernández-Hidalgo N., Cuéllar-Calabria H., Domènech A.P., Gonzàlez-Alujas M.T., Subirana-Domènech M.T., Miranda-Barrio B., Ferreira-González I. (2017). 18F-FDG-PET/CT angiography in the diagnosis of infective endocarditis and cardiac device infection in adult patients with congenital heart disease and prosthetic material. Int. J. Cardiol..

[57] Saby L., Habib G., Laas O., Cammilleri S., Casalta J.P., Gouriet F., Riberi A., Avierinos J.F., Raoult D., Thuny F. (2013). Positron emission tomography/computed tomography for diagnosis of prosthetic valve endocarditis: Increased valvular 18F-fluorodeoxyglucose uptake as a novel major criterion. J. Am. Coll. Cardiol..

[58] Rouzet F., Chequer R., Benali K., Lepage L., Ghodbane W., Duval X., Iung B., Vahanian A., Le Guludec D., Hyafil F. (2014). Respective performance of 18F-FDG PET and radiolabeled leukocyte scintigraphy for the diagnosis of prosthetic valve endocarditis. J. Nucl. Med..

[59] Ricciardi A., Sordillo P., Ceccarelli L., Maffongelli G., Calisti G., Di Pietro B., Caracciolo C.R., Schillaci O., Pellegrino A., Chiariello L. (2014). 18-Fluoro-2-deoxyglucose positron emission tomography-computed tomography: An additional tool in the diagnosis of prosthetic valve endocarditis. Int. J. Infect. Dis..

[60] Bartoletti M., Tumietto F., Fasulo G., Giannella M., Cristini F., Bonfiglioli R., Raumer L., Nanni C., Sanfilippo S., Di Eusanio M. (2014). Combined computed tomography and fluorodeoxyglucose positron emission tomography in the diagnosis of prosthetic valve endocarditis: A case series. BMC Res. Notes.

[61] Roque A., Pizzi M.N., Fernández-Hidalgo N., Permanyer E., Cuellar-Calabria H., Romero-Farina G., Ríos R., Almirante B., Castell-Conesa J., Escobar M. (2020). Morpho-metabolic post-surgical patterns of non-infected prosthetic heart valves by [18F]FDG PET/CTA: “normality” is a possible diagnosis. Eur. Heart J. Cardiovasc. Imaging.

[62] Wahadat A.R., Tanis W., Scholtens A.M., Bekker M., Graven L.H., Swart L.E., den Harder A.M., Lam M.G., De Heer L.M., Roos-Hesselink J.W. (2021). Normal imaging findings after aortic valve implantation on ^18^F-Fluorodeoxyglucose positron emission tomography with computed tomography. J. Nucl. Cardiol..

[63] Wahadat A.R., Tanis W., Swart L.E., Scholtens A., Krestin G.P., van Mieghem N.M.D.A., Schurink C.A.M., van der Spoel T.I.G., Brink F.S.v.D., Vossenberg T. (2021). Added value of ^18^F-FDG-PET/CT and cardiac CTA in suspected transcatheter aortic valve endocarditis. J. Nucl. Cardiol..

[64] San S., Abulizi M., Moussafeur A., Oliver L., Lepeule R., Nahory L., Faivre L., Huguet R., Nguyen A., Gallien S. (2021). Characterization of 18-fluorodeoxyglucose uptake pattern in infective endocarditis after transcatheter aortic valve implantation. Eur. Heart J. Cardiovasc. Imaging.

[65] Lucinian Y.A., Lamarche Y., Demers P., Martineau P., Harel F., Pelletier-Galarneau M. (2020). FDG-PET/CT for the Detection of Infection Following Aortic Root Replacement Surgery. JACC Cardiovasc. Imaging.

[66] Cabell C.H., A Heidenreich P., Chu V.H., Moore C.M., E Stryjewski M., Corey G., Fowler V.G. (2004). Increasing rates of cardiac device infections among Medicare beneficiaries: 1990–1999. Am. Heart J..

[67] Greenspon A.J., Patel J.D., Lau E., Ochoa J.A., Frisch D.R., Ho R.T., Pavri B.B., Kurtz S.M. (2011). 16-year trends in the infection burden for pacemakers and implantable cardioverter-defibrillators in the United States 1993 to 2008. J. Am. Coll. Cardiol..

[68] Mahmood M., Kendi A.T., Ajmal S., Farid S., O’horo J.C., Chareonthaitawee P., Baddour L.M., Sohail M.R. (2019). Meta-analysis of 18F-FDG PET/CT in the diagnosis of infective endocarditis. J. Nucl. Cardiol..

[69] Juneau D., Golfam M., Hazra S., Erthal F., Zuckier L.S., Bernick J., Wells G.A., Beanlands R.S., Chow B.J. (2018). Molecular Imaging for the diagnosis of infective endocarditis: A systematic literature review and meta-analysis. Int. J. Cardiol..

[70] Ploux S., Riviere A., Amraoui S., Whinnett Z., Barandon L., Lafitte S., Ritter P., Papaioannou G., Clementy J., Jais P. (2011). Positron emission tomography in patients with suspected pacing system infections may play a critical role in difficult cases. Heart Rhythm..

[71] Hove D.T., Treglia G., Slart R.H.J.A., Damman K., Wouthuyzen-Bakker M., Postma D.F., Gheysens O., Borra R.J.H., Mecozzi G., van Geel P.P. (2021). The value of 18F-FDG PET/CT for the diagnosis of device-related infections in patients with a left ventricular assist device: A systematic review and meta-analysis. Eur. J. Nucl. Med. Mol. Imaging.

[72] Kim J., Feller E.D., Chen W., Liang Y., Dilsizian V. (2019). FDG PET/CT for early detection and localization of left ventricular assist device infection: Impact on patient management and outcome. JACC Cardiovasc. Imaging.

[73] Sohns J.M.S., Kroehn H., Schoede A., Derlin T., Haverich A., Schmitto J.D., Bengel F.M. (2020). 18 F-FDG PET/CT in left-ventricular assist device infection: Initial results supporting the usefulness of image-guided therapy. J. Nucl. Med..

[74] Godefroy T., Frécon G., Asquier-Khati A., Mateus D., Lecomte R., Rizkallah M., Piriou N., Jamet B., Le Tourneau T., Pallardy A. (2023). ^18^F-FDG-Based Radiomics and Machine Learning: Useful Help for Aortic Prosthetic Valve Infective Endocarditis Diagnosis?. JACC Cardiovasc. Imaging.

[75] Auletta S., Varani M., Horvat R., Galli F., Signore A., Hess S. (2019). PET Radiopharmaceuticals for Specific Bacteria Imaging: A Systematic Review. J. Clin. Med..

[76] Weinstein E.A., Ordonez A.A., DeMarco V.P., Murawski A.M., Pokkali S., MacDonald E.M., Klunk M., Mease R.C., Pomper M.G., Jain S.K. (2014). Imaging enterobacteriaceae infection in vivo with 18F-fluorodeoxysorbitol positron emission tomography. Sci. Transl. Med..

[77] Gowrishankar G., Hardy J., Wardak M., Namavari M., Reeves R.E., Neofytou E., Srinivasan A., Wu J.C., Contag C.H., Gambhir S.S. (2017). Specific Imaging of Bacterial Infection Using 6″-18F-Fluoromaltotriose: A Second-Generation PET Tracer Targeting the Maltodextrin Transporter in Bacteria. J. Nucl. Med..

[78] Mutch C.A., Ordonez A.A., Qin H., Parker M., Bambarger L.E., Villanueva-Meyer J.E., Blecha J., Carroll V., Taglang C., Flavell R. (2018). [^11^C]Para-Aminobenzoic Acid: A Positron Emission Tomography Tracer Targeting Bacteria-Specific Metabolism. ACS Infect. Dis..

[79] Ordonez A.A., Weinstein E.A., Bambarger L.E., Saini V., Chang Y.S., DeMarco V.P., Klunk M.H., Urbanowski M.E., Moulton K.L., Murawski A.M. (2017). A systematic approach for developing bacteria-specific imaging tracers. J. Nucl. Med..

[80] Zhang Z., Ordonez A.A., Smith-Jones P., Wang H., Gogarty K.R., Daryaee F., Bambarger L.E., Chang Y.S., Jain S.K., Tonge P.J. (2017). The biodistribution of 5-[18F] fluoropyrazinamide in Mycobacterium tuberculosis-infected mice determined by positron emission tomography. PLoS ONE.

